# DNA fluctuations reveal the size and dynamics of topological domains

**DOI:** 10.1093/pnasnexus/pgac268

**Published:** 2022-11-22

**Authors:** Willem Vanderlinden, Enrico Skoruppa, Pauline J Kolbeck, Enrico Carlon, Jan Lipfert

**Affiliations:** Department of Physics and Center for NanoScience (CeNS), LMU Munich, Amalienstrasse 54, 80799 Munich, Germany; Department of Physics and Debye Institute for Nanomaterials Science, Utrecht University, Princetonplein 1, 3584 CC Utrecht, The Netherlands; Soft Matter and Biophysics, Department of Physics and Astronomy, KU Leuven, Celestijnenlaan 200D, 3001 Leuven, Belgium; Department of Physics and Center for NanoScience (CeNS), LMU Munich, Amalienstrasse 54, 80799 Munich, Germany; Department of Physics and Debye Institute for Nanomaterials Science, Utrecht University, Princetonplein 1, 3584 CC Utrecht, The Netherlands; Soft Matter and Biophysics, Department of Physics and Astronomy, KU Leuven, Celestijnenlaan 200D, 3001 Leuven, Belgium; Department of Physics and Center for NanoScience (CeNS), LMU Munich, Amalienstrasse 54, 80799 Munich, Germany; Department of Physics and Debye Institute for Nanomaterials Science, Utrecht University, Princetonplein 1, 3584 CC Utrecht, The Netherlands

**Keywords:** DNA, DNA topology, magnetic tweezers, single-molecule methods

## Abstract

DNA supercoiling is a key regulatory mechanism that orchestrates DNA readout, recombination, and genome maintenance. DNA-binding proteins often mediate these processes by bringing two distant DNA sites together, thereby inducing (transient) topological domains. In order to understand the dynamics and molecular architecture of protein-induced topological domains in DNA, quantitative and time-resolved approaches are required. Here, we present a methodology to determine the size and dynamics of topological domains in supercoiled DNA in real time and at the single-molecule level. Our approach is based on quantifying the extension fluctuations—in addition to the mean extension—of supercoiled DNA in magnetic tweezers (MT). Using a combination of high-speed MT experiments, Monte Carlo simulations, and analytical theory, we map out the dependence of DNA extension fluctuations as a function of supercoiling density and external force. We find that in the plectonemic regime, the extension variance increases linearly with increasing supercoiling density and show how this enables us to determine the formation and size of topological domains. In addition, we demonstrate how the transient (partial) dissociation of DNA-bridging proteins results in the dynamic sampling of different topological states, which allows us to deduce the torsional stiffness of the plectonemic state and the kinetics of protein-plectoneme interactions. We expect our results to further the understanding and optimization of magnetic tweezer measurements and to enable quantification of the dynamics and reaction pathways of DNA processing enzymes in the context of physiologically relevant forces and supercoiling densities.

Significance Statement In the cell, long DNA molecules carry the genetic information and must be stored and maintained yet remain accessible for readout and processing. DNA supercoiling facilitates the compaction of DNA, modulates its accessibility, and spatially juxtaposes DNA sites distant in linear DNA sequence. By binding to two sites in supercoiled DNA, DNA bridging proteins can pinch off topological domains and alter DNA plectoneme dynamics. Here, we show how DNA bridging and topological domain dynamics can be detected from changes in the extension fluctuations of supercoiled DNA molecules tethered in magnetic tweezers. Our work highlights how considering DNA extension fluctuations in addition to the mean extension, provides additional information and enables the investigation of protein-DNA interactions that are otherwise invisible.

## Introduction

**Fig. 1.**
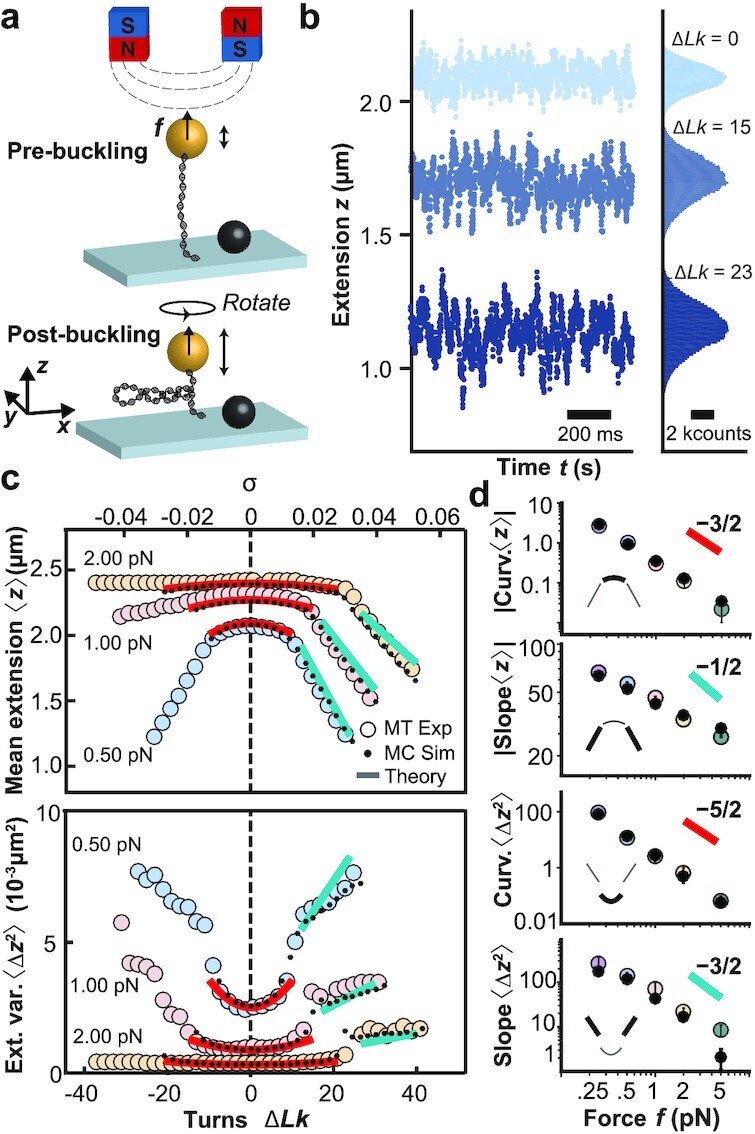
DNA extension fluctuation as a function of linking number and applied force. (a) Schematic of MT experiment applying forces and controlling the linking number of a DNA molecule tethered between a flow cell surface and a magnetic bead. (b) Time traces of experimentally measured extension *z* for three linking number differences Δ*Lk*. The data show a decrease in 〈*z*〉 and an increase in 〈Δ*z*^2^〉 when Δ*Lk* is increased from the torsionally relaxed state Δ*Lk* = 0. (c) MT experimental data for 〈*z*〉 and 〈Δ*z*^2^〉 vs. Δ*Lk* (or alternatively vs. the supercoiling density, σ ≡ Δ*Lk*/*Lk*_0_, top axis) for three different forces, *f* = 0.5, 1, and 2 pN (large, colored circles). The small black circles and solid lines are predictions from Monte Carlo simulation and the analytical theories (see main text), respectively. (d) Curvatures in the prebuckling and of slopes in the postbuckling regimes for 〈*z*〉 and 〈Δ*z*^2^〉 vs. applied force, *f*. The data are shown in double-logarithmic representation. Large colored circles are the mean ± std from at least eight independent measurements. Black dots are from Monte Carlo simulations, where error bars represent the uncertainty of the fit. According to the Moroz and Nelson (MN) model ((43) and Supplementary Material), the prebuckling curvatures of 〈*z*〉 and 〈Δ*z*^2^〉 are expected to scale as *f*^−3/2^ and *f*^−5/2^ for large forces, respectively. The postbuckling slopes of 〈*z*〉 and 〈Δ*z*^2^〉 are predicted to scale as *f*^−1/2^ and *f*^−3/2^.

Genomic DNA is highly compacted for efficient storage but must be made transiently accessible to facilitate the readout and processing of the genetic material (1,2). A central mechanism to balance compaction and local accessibility is DNA supercoiling. In the cell, DNA is maintained in an underwound state and can adopt plectonemic conformations, i.e., highly entangled structures consisting of intertwined superhelices (3–8). Importantly, plectonemes spatially juxtapose distant sites in linear DNA sequence and, therefore, enable the bridging and looping of DNA by proteins that engage multiple binding sites. Protein-induced conformational changes and separation of topological domains in DNA, in turn, provide another critical level of genomic regulation (6,9–15). A large body of experimental and computational research has resulted in a quantitative understanding of the average geometry of supercoiled DNA under a range of environmental conditions (7,15–23). In particular, single-molecule magnetic tweezers (MT) have provided a powerful tool to study DNA under precisely controlled levels of applied force and supercoiling (16,21,24,25) by tethering DNA molecules between a surface and small, magnetic beads (Fig. 1a). Using external magnets, calibrated stretching forces are applied, and the DNA linking number, *Lk*, is controlled by rotating the magnets. MT have provided a wealth of information about the mechanical properties of DNA (16,18) and about DNA processing enzymes, including polymerases (26–28), topoisomerases (24,29–31), gyrases (32), and other DNA binding proteins (33–36). In MT experiments, typically, the extension of the DNA tether, *z*, is followed as a function of time. The resulting average extension, 〈*z*〉, of the DNA in response to applied forces and imposed linking difference Δ*Lk* has been studied extensively both experimentally by MT and theoretically, and is well understood (16,19,22,24,37–39). Here, we focus on the variance of the extension, 〈Δ*z*^2^〉, and show that by analyzing extension fluctuations in addition to the mean extension, we can quantify the size and dynamics of topological domains.

We first perform high-speed MT measurements to map out in detail the level of fluctuations of DNA as a function of applied force and linking number. We show that the extension fluctuations can be understood semiquantitatively by an analytical two-state model that describes DNA as an isotropic elastic rod with a straight and a plectonemic phase. We then present Monte Carlo simulations of the DNA chain that are in quantitative agreement with experiments and provide microscopic insight into the origin of the fluctuations. Using this theoretical framework, we show how changes in fluctuations enable the monitoring of protein-induced bridging in supercoiled DNA. We validate predictions of our model experimentally by observing DNA fluctuation changes upon binding of two-site restriction enzymes to DNA. Last, we demonstrate the possibility of quantifying the dynamics of transient, partial dissociation, and the energy penalty of trapping loops with different supercoiling densities.

## Results

### Extension fluctuations of supercoiled DNA under tension

We performed systematic MT experiments on 7.9 kbp DNA molecules tethered between a flow cell surface and 1.0 µm-diameter magnetic beads under well-defined forces and linking differences (Δ*Lk* = *Lk* − *Lk*_0_, the difference in linking number *Lk* relative to the torsionally relaxed molecule with *Lk*_0_; Fig. 1a). We use a custom-build MT instrument (Fig. 1a and "Methods" section) and high-speed tracking at 1 kHz to accurately capture fast fluctuations. Time traces of the DNA tether extension at a constant applied stretching force, *f*, reveal systematic changes of both the mean and variance of the extension as a function of applied turns, i.e., at different Δ*Lk* (Fig. 1b).

From the extension time traces, we obtain data of mean extension, 〈*z*〉, and extension variance, 〈Δ*z*^2^〉, as functions of Δ*Lk* and *f* (Fig. 1c). For sufficiently small *f*, the response in both 〈*z*〉 and 〈Δ*z*^2^〉 is symmetric for over- (Δ*Lk* > 0) or underwinding (Δ*Lk* < 0). At *f* ≥ 1 pN the DNA response becomes asymmetric due to torque-induced melting upon underwinding (16,40–42). Here, we focus on overwound DNA, Δ*Lk* > 0, i.e., the regime where the DNA remains double-stranded. Overall, 〈*z*〉 decreases with increasing Δ*Lk*, while 〈Δ*z*^2^〉 increases (Fig. 1c,d), with two different regimes: at small Δ*Lk* the mean extension decreases only slowly with Δ*Lk*, which is the prebuckling regime in which the DNA is stretched and extended (Fig. 1c and d regions with red line fits). Beyond a characteristic force-dependent Δ*Lk*, the molecule buckles and undergoes a conformational transition into a partially plectonemic state, i.e., a portion of the molecule assumes interwound configurations of the double-helix axis (Fig. 1a bottom and Fig. 1c and d regions with light blue line fits). In this postbuckling regime, an increase of Δ*Lk* leads to an increase in the size of the plectoneme, causing a linear decrease of 〈*z*〉 with increasing Δ*Lk*. The dependence of 〈*z*〉 on Δ*Lk* and force has been extensively studied experimentally and described by a number of different models (43–50).

The characteristic timescales of the fluctuations obtained from temporal autocorrelation analysis, overall, show a very similar behavior as 〈Δ*z*^2^〉, increasing first quadratically and then linearly with increasing Δ*Lk* and also increasing with decreasing force (Fig. S1). For the range of conditions investigated here, the characteristic times are >1 ms, such that our measurements at 1 kHz provide sufficient sampling.

### Analytical models for extension fluctuations of supercoiled DNA

Here, we extend previous analyses to account for the dependence of the variance, 〈Δ*z*^2^〉, on *f* and Δ*Lk* for a DNA molecule of length *L*. Mean and variance are obtained by differentiating the free energy per unit length $\mathcal {F}$ with respect to the force, *f*,

(1) $$\begin{eqnarray*}
\frac{\langle z \rangle }{L} &= - \frac{d {\mathcal {F}}}{df},
\end{eqnarray*}
$$

(2) $$\begin{eqnarray*}
\frac{\langle \Delta z^2 \rangle }{L} &= - k_B T\frac{d^2 {\mathcal {F}}}{df^2} = \frac{k_B T}{L} \frac{d{\langle z \rangle }}{df},
\end{eqnarray*}
$$

with *k_B_* the Boltzmann constant and *T* the temperature. Eqs. (1) and (2) follow from equilibrium statistical mechanics (see Supplementary Material Section 2A for details) and imply that 〈*z*〉 and 〈Δ*z*^2^〉 must have the same functional dependence on Δ*Lk*.

The theory by Moroz and Nelson (MN) describes the response of DNA in the prebuckling regime (43,44) and predicts that 〈*z*〉 –and as a consequence of Eq. (2) also 〈Δ*z*^2^〉– varies quadratically with Δ*Lk*. The prediction of the MN model, using accepted values for the bending persistence length, *A*, and twist persistence length, *C*, (*A* = 40 nm, and *C* = 100 nm; see Fig. S2 and (22,51)), semiquantitatively reproduces the linking number and force-dependent trends of both the measured 〈*z*〉 and 〈Δ*z*^2^〉 in the prebuckling regime (Fig. 1c, red lines). In particular, the quadratic dependence of 〈*z*〉 on Δ*Lk* extends to a quadratic dependence of 〈Δ*z*^2^〉 as predicted by Eq. (2). Explicit expressions derived from the MN model for 〈Δ*z*^2^〉 are given in the Supplementary Material (Eqs. 10 and 11).

To extend the analysis into the postbuckling regime, we employ the two-phase model by Marko (45). We note that Bouchiat and Mezard (52) have previously computed 〈Δ*z*^2^〉 by mapping the twisted rod model into a quantum mechanical problem. However, we follow the approach by Marko that enables a more straight-forward derivation of expressions for the changes of 〈*z*〉 and 〈Δ*z*^2^〉, which is relevant for the analysis of protein-DNA interactions. For convenience, we use the supercoiling density, σ = Δ*Lk*/*Lk*_0_, instead of the DNA length-dependent Δ*Lk*. In the two-phase model, a DNA molecule is considered to be composed of two different phases, a stretched and a plectonemic phase, which are governed by distinct free energies per unit length, ${\mathcal {S}}(\sigma )$ and ${\mathcal {P}}(\sigma )$, (expressions for ${\mathcal {S}}$ and ${\mathcal {P}}$ are given in Supplementary Material, Section 2B). For supercoiling densities below the critical value, σ_*s*_, the molecule is fully in the stretched phase. At the buckling point, σ = σ_*s*_, the molecule undergoes a pseudo-first-order phase transition (53) and separates into stretched and plectonemic phases with supercoiling densities σ_*s*_ and σ_*p*_, respectively. Finally, for large supercoiling densities, σ > σ_*p*_, the molecule fully assumes the plectonemic phase, where the two ends of the molecule are in close vicinity and the extension vanishes. The full plectonemic phase is, however, difficult to probe in MT, due to steric repulsion between the bead, DNA, and the surface, and we focus here on the prebuckling and coexistence regimes.

Estimates of σ_*s*_ and σ_*p*_ can be obtained, in principle, from specific statistical polymer models (45–50). However, we focus here on the universal properties of the two-phase model that are independent of specific values of σ_*s*_ and σ_*p*_. In the coexistence region, σ_*s*_ < σ < σ_*p*_, the free energy of the molecule is obtained by a thermodynamic double tangent construction, which gives a free energy linear in σ (46),

(3) $$\begin{eqnarray*}
{\mathcal {F}}(\sigma ) = \frac{\sigma _p - \sigma }{\sigma _p - \sigma _s} \, {\mathcal {S}}(\sigma _s) + \frac{\sigma - \sigma _s}{\sigma _p -\sigma _s} \, {\mathcal {P}}(\sigma _p),
\end{eqnarray*}
$$

where (σ_*p*_ − σ)/(σ_*p*_ − σ_*s*_) ≡ ν and (σ − σ_*s*_)/(σ_*p*_ − σ_*s*_) = 1 − ν are the average fractions of the DNA in the stretched and plectonemic phases, respectively. The two coexisting phases have average supercoiling densities σ_*s*_ and σ_*p*_ and free energies per unit length ${\mathcal {S}}(\sigma _s)$ and ${\mathcal {P}}(\sigma _p)$. The average extension according to Eq. (1) assumes the form

(4) $$\begin{eqnarray*}
\frac{\langle z \rangle }{L} = \Gamma \left( \sigma _p - \sigma \right),
\end{eqnarray*}
$$

where Γ is a force-dependent prefactor giving the slope of 〈*z*〉/*L* vs. σ in the postbuckling regime. The linear dependence on σ is a consequence of the linearity of the double tangent construction for the free energy (3). Eq. (4) can be understood as follows: at phase coexistence, σ_*s*_ < σ < σ_*p*_, the average fraction in the stretched phase is given by ν = (σ_*p*_ − σ)/(σ_*p*_ − σ_*s*_), where ν = 1 at σ = σ_*s*_ and ν = 0 at σ = σ_*p*_. Only the stretched phase contributes to the average extension, hence 〈*z*〉 must be proportional to ν, which leads to Eq. (4). Using Eq. (2), the extension variance is obtained by differentiation of Eq. (4) with respect to *f*

(5) $$\begin{eqnarray*}
\frac{\langle \Delta z^2 \rangle }{L} = k_B T \left( \frac{\partial \Gamma }{\partial f} \left( \sigma _p - \sigma \right) + \Gamma \frac{\partial \sigma _p}{\partial f} \right),
\end{eqnarray*}
$$

as both the proportionality constant Γ and σ_*p*_ depend on the applied force (46). From basic phenomenological considerations (see Supplementary Material Section 2A) one can deduce that ∂Γ/∂*f* < 0, which implies a linear increase of 〈Δ*z*^2^〉 with σ. An increase of the variance with σ is consistent with previous theoretical considerations (52) and with force-extension measurements of supercoiled DNA, which revealed a softer elastic response with increasing supercoiling density for positive supercoiled DNA (16). As the variance, 〈Δ*z*^2^〉, is given by differentiation of 〈*z*〉 with respect to *f* (Eq. 2), a softer response with increasing σ implies an increase of 〈Δ*z*^2^〉. Analytical approximations for 〈*z*〉 and 〈Δ*z*^2^〉 can be obtained by employing a quadratic free energy for the plectonemic state as a function of the supercoiling density σ with an associated effective torsional stiffness, *P* (46) (see Supplementary Supplementary Material Section 2B). This model captures the general trends of the experimental data, including the rapid increase of the variance at the buckling transition and the linear increase in the postbuckling regime.

The scaling relationships indicated in Fig. 1d also accurately predict the trends of the signal-to-noise ratio for single-molecule measurements of DNA topology with force (Fig. S3). However, the two-phase model fails to accurately capture the slopes in the postbuckling regime (Fig. 1c, turquoise lines). The disagreement may be due to the approximations introduced in the estimate of the plectoneme free energy or due to the limitations of the two-state approximation (20,46,49,50,54,55).

We note that the characteristic timescales of the fluctuations, τ_*c*_, have essentially the same dependencies on Δ*Lk* and *f* as the variance, 〈Δ*z*^2^〉 (Fig. S1). This is expected, as for a harmonic potential, the characteristic timescales are related to the variance by τ_*c*_ = (γ/*k_B_T*) · 〈Δ*z*^2^〉, where*γ* is the friction coefficient.

### Monte Carlo simulations quantitatively capture DNA extension fluctuations

**Fig. 2.**
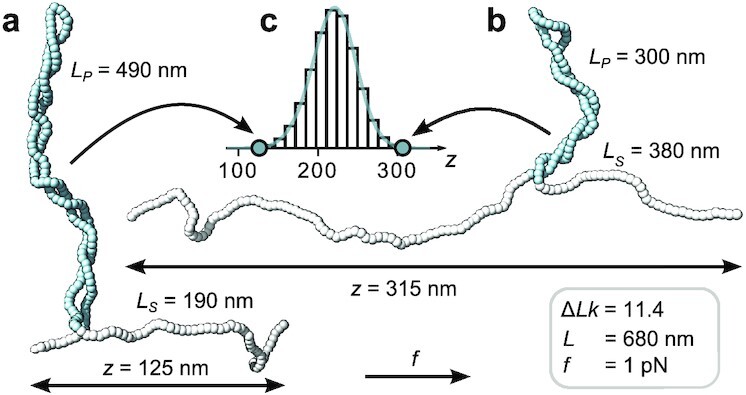
Monte Carlo simulations reveal extension fluctuation due to exchange of DNA length between the stretched and plectonemic states. (a,b) Snapshots of torsionally constrained and stretched linear DNA molecules in the post-buckling regime generated by a MC simulation (see "Methods" section). The molecule has a total length of *L* = 680 nm and is subjected to a stretching force of *f* = 1 pN while being maintained at fixed linking number Δ*Lk* = 11.4 (corresponding to σ = 0.06). (c) Extension distribution from a single simulation run. The molecular configurations in (a) and (b) were chosen from opposite tails of the extension distribution in (c). In the low extension configuration (a) a significantly larger amount of contour length (*L_p_* = 490 nm, blue segments) is contained in the plectonemic phase than in the high extension configuration (b), which exhibits a much more pronounced stretched phase (*L_s_* = 380 nm, white segments). The simulations suggest that the exchange of DNA length between the plectonemic and stretched phases gives a major contribution to 〈Δ*z*^2^〉.

To quantitatively describe the experimental data and to provide microscopic insights into the origin of the postbuckling fluctuations, we carried out Monte Carlo (MC) simulations ("Methods" section and Figure S4). The simulations are based on a discretization of the self-avoiding twistable wormlike chain model, and we use the same values for the elastic parameters, *A* and *C*, as in the analytical models above. The MC simulations provide an excellent description of the experimentally determined 〈*z*〉 and 〈Δ*z*^2^〉 values (Fig. 1c, small black circles) and also capture the correct force dependencies of the curvatures of 〈*z*〉 and 〈Δ*z*^2^〉 in the prebuckling regime and the slopes in the postbuckling regime (Fig. 1d, small black circles, and Table S1). In addition, the MC simulations give access to the microscopic conformations of the DNA chain, which provides an intuitive explanation of what drives the increase in extension fluctuations in the postbuckling regime. In this regime, the total DNA length, *L*, partitions into length in the stretched state, *L_s_*, and length in the plectonemic state, *L_p_*, where *L* = *L_s_* + *L_p_*, but only *L_s_* contributes to the tether extension. The exchange of DNA length between the two states, therefore, leads to pronounced extension fluctuations (Fig. 2). In particular, the rapid increase of 〈Δ*z*^2^〉 at the buckling point stems from the onset of these exchange fluctuations. As σ is further increased in the postbuckling regime, the average length of the plectonemic phase increases. Since the plectonemic phase is torsionally softer than the stretched phase, its growth leads to an increase of torsional fluctuations, which in turn results in an increase of fluctuations in *z*.

### DNA bridging proteins reduce extension fluctuations, but not mean extension

Proteins that interact via two DNA sites (e.g., recombinases, transcription factors, architectural proteins, or many restriction enzymes) can bridge across plectonemic segments and thereby introduce DNA topological domains (12,14,56,57) (Fig. S5). Here, we demonstrate that DNA-bridging proteins induce a reduction in 〈Δ*z*^2^〉, but do not modify 〈*z*〉 on average. We develop a simple theoretical description based on two assumptions: (i) the protein bridges two DNA sites in the plectonemic region and generates a loop that constitutes a topological domain of length Δ*L* (referred to as looped DNA), which does not significantly interact with the rest of the molecule; (ii) the remaining DNA of length, L* ≡ *L* − Δ*L* (referred to as unlooped DNA), can be described by the two-phase model (46) via Eqs. (4) and (5). Using these two assumptions, one can estimate 〈*z*〉* and 〈Δ*z*^2^〉*, the equilibrium values of the average extension and of the extension variance of the DNA with a protein bound. The linking number of the unlooped DNA is obtained by subtracting from the total Δ*Lk* the contribution “trapped” in the looped part, which is in the plectoneme characterized by a supercoiling density σ_*p*_. The supercoiling density of the unlooped DNA, σ*, is hence given by σ*L* = σ*L* − σ_*p*_Δ*L* and, using Δ*L* = *L* − L*, one obtains L*(σ_*p*_ − σ*) = *L*(σ_*p*_ − σ). Substituting the latter in Eq. (4) one obtains 〈*z*〉* = 〈*z*〉, i.e., the average extension does not change upon protein binding. Using the same relation in Eq. (5) we find

(6) $$\begin{eqnarray*}
\Delta \langle \Delta z^2 \rangle \equiv \langle \Delta z^2 \rangle ^{*} - \langle \Delta z^2 \rangle = - k_B T \, \Gamma \frac{\partial \sigma _p}{\partial f} \, \Delta L.
\end{eqnarray*}
$$

Therefore, upon protein-induced bridging, the variance is predicted to decrease by an amount proportional to the looped DNA length, Δ*L*. This result can be understood by considering that in bare DNA, the fluctuations in *z* at postbuckling are predominantly due to the exchange of length between stretched and plectonemic phases (Fig. 2). This length exchange is suppressed by the presence of a bridging protein, which prevents the plectoneme from becoming shorter than Δ*L*. In fact, while the extension in bare DNA is bounded by *z* ≤ *L* for a DNA with a looped part of length Δ*L*, the extension is bounded by *z* ≤ *L* − Δ*L*. The proportionality factor linking Δ*L* and Δ〈Δ*z*^2^〉 in Eq. (6) can be obtained from the variance of bare DNA Eq. (5) in the limit σ → σ_*p*_

(7) $$\begin{eqnarray*}
\frac{1}{L} \lim _{\sigma \rightarrow \sigma _p} \langle \Delta z^2 \rangle = k_B T \Gamma \frac{\partial \sigma _p}{\partial f}.
\end{eqnarray*}
$$

**Fig. 3.**
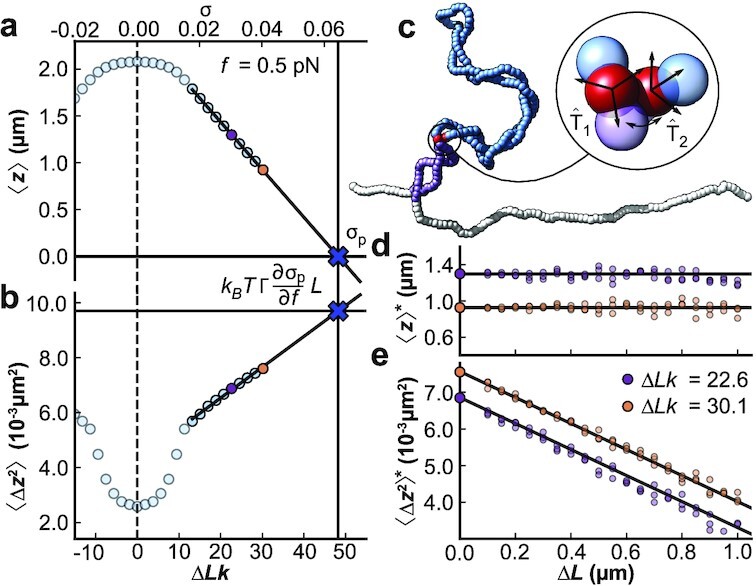
Monte Carlo simulations show the effect of DNA bridging proteins on average extension and extension fluctuations. (a,b) 〈*z*〉 and 〈Δ*z*^2^〉 vs. Δ*Lk* (and σ, top axis) from MC simulations for a 7.9 kbp DNA molecule at *f* = 0.5 pN. The solid lines are fits for the extrapolation to deduce σ_*p*_ (in panel a) and the proportionality factor $k_B T \Gamma \frac{\partial \sigma _p}{\partial f}$ (in panel b), respectively. Using this extrapolation scheme, we can predict 〈Δ*z*^2^〉*, the variance after a protein-bridging event. (c) Snapshot of a constrained MC simulation at σ = 0.04 mimicking the effect of a protein binding at the two sites indicated by red beads. Throughout the simulation, the distance and relative orientation of the two beads is kept fixed, effectively partitioning the DNA molecule into a looped part of length Δ*L* (dark blue) and an unlooped part of length L* = *L* − Δ*L* (white and purple). (d,e) MC data of 〈*z*〉* and 〈Δ*z*^2^〉*, the values of the average extension and extension variance after protein binding, for σ = 0.03 (purple) and σ = 0.04 (yellow). While 〈*z*〉* does not change with Δ*L* (d), 〈Δ*z*^2^〉* is linearly dependent on Δ*L* (e), in agreement with the predictions of our model (see main text). The horizontal line in (d) indicates 〈*z*〉 for a DNA with no proteins bound. The intercept of the solid line in (e) is set to the free DNA value of 〈Δ*z*^2^〉, and its slope is determined from the extrapolation scheme in panels (a) and (b). All data points shown are calculated from ensembles generated by ten independent MC simulations, run for 10^9^ iteration steps each, and sampled every 1,000 steps. Error bars are smaller than the symbols. The three different points in (d) and (e) for the same conditions correspond to three separate configurations implementing loops of a given size.

The proportionality in Eq. (6) (i.e., the right-hand side of Eq. 7) can be found by extrapolation. First, we determine the plectonemic supercoiling density, σ_*p*_, by linearly extrapolating the extension in the postbuckling regime to zero (Fig. 3a see also Eq. 4). We note that either MC data (Fig. 3a) or experimental data (Fig. S6a) can be used for this extrapolation. Second, we then extrapolate 〈Δ*z*^2^〉 in the regime where it increases linearly with σ to σ_*p*_, which again can employ either MC or experimental data (Fig. 3b and Fig. S6b, see also Eq. 5). Extrapolation is necessary since the extension does not decrease all the way to zero due to finite size effects and the presence of the surface.

We test the relation between Δ*L* and 〈Δ*z*^2^〉 (Eq. 6) in our Monte Carlo simulations by imposing an effective bridging between two segments on a plectomene. Starting from equilibrated snapshots of DNA simulations for two different σ (0.03 and 0.04) at constant force (*f* = 0.5 pN), potential binding sites were selected based on a distance threshold of 8 nm between two coarse-grained beads located on opposite strands of a single plectoneme. The effect of protein binding was then mimicked by fixing the relative position and orientation of sites chosen to generate looped domains of particular lengths Δ*L* (illustrated by the red beads of Fig. 3c) in the further simulation. By repeating simulations with different bridging sites, we determine the average extension and variance, 〈*z*〉* and 〈Δ*z*^2^〉*, after the constraint is introduced vs. looped DNA length, Δ*L* (Fig. 3d and e, and Figure S7). The MC simulations reproduce the predictions of our model, with 〈*z*〉* essentially unaffected and 〈Δ*z*^2^〉* decreasing linearly with Δ*L*. We stress that the solid lines in Fig. 3e are not a fit but a direct prediction of our model using the extrapolation schemes to determine σ_*p*_ (Fig. 3a) and the proportionality factor via Eq. (7) (Fig. 3b).

**Fig. 4.**
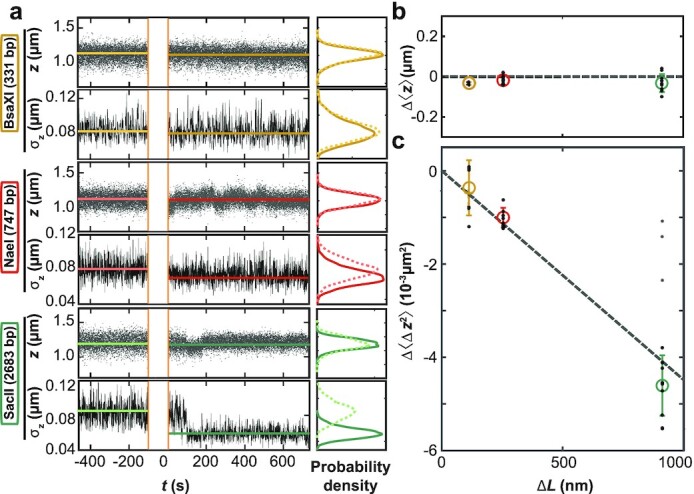
Effect of DNA bridging restriction enzymes on 〈*z*〉 and 〈Δ*z*^2^〉 in MT. (a) Time traces for *z* and σ_*z*_ = (〈Δ*z*^2^〉)^1/2^ measured in MT before and after addition of restriction enzymes at time *t* = 0. The restriction enzymes used are indicated on the left. Data for *z* are raw data recorded at 1 kHz. The standard deviation σ_*z*_(*t*) was computed from *z*(*t*) using 1 s intervals. The histograms show the distribution of data points before (dashed lines) and after (solid lines) introduction of the proteins. (b) Change in mean extension Δ〈*z*〉 after protein binding as a function of expected loop length Δ*L* (Table 1). (c) Change in extension variance Δ〈Δ*z*^2^〉 after protein binding vs. Δ*L*. The data in (b) and (c) are from at least five independent measurements for each enzyme. We quantified 〈Δ*z*^2^〉 using the mean of the variance distributions. Black dots are individual measurements, colored circles and error bars the mean ± std over the independent measurements. The data show no substantial change in 〈*z*〉 and a drop in 〈Δ*z*^2^〉 proportional to Δ*L* in agreement with Eq. (6), which is shown as a dashed line that is not a fit, but obtained from extrapolation of the experimental data (Fig. S6). A fraction of the experimental data points for the enzyme SacII deviate from the expected behavior (gray dots; excluded from further analysis), possibly indicating an alternative binding mode. All data shown were obtained at *f* = 0.5 pN and σ = 0.04.

To test the effect of DNA bridging proteins on 〈*z*〉* and 〈Δ*z*^2^〉* experimentally, we used three different two-site DNA restriction enzymes that are sequence-specific and possess only one pair of binding sites along the DNA construct used in our MT experiments ("Methods" section and Table 1). We impede enzymatic cleavage by using Ca^2+^ instead of Mg^2+^ in the reaction buffer and work under conditions where ${\lt }30\%$ of tethers show signs of enzyme binding, such that it is unlikely for one DNA molecule to interact with multiple enzymes. In the experiments, we first introduce positive supercoils and record the molecular extension as a function of time in the absence of protein. Subsequently, we introduce the proteins in the flow cell and again obtain extension time traces (Fig. 4a and Fig. S8). We find that the mean extensions, 〈*z*〉, remain essentially unaltered upon addition of the DNA-bridging proteins (Fig. 4a and b). In contrast, the variance (or equivalently the standard deviation) of the extension fluctuations, 〈Δ*z*^2^〉, computed from the experimental trace using a 1 s time window, decreases upon protein binding (Fig. 4a and c). The observed decrease of the variance, 〈Δ*z*^2^〉, is in good agreement with the prediction of our model (Eq. 6). The dashed line in Fig. 4c is again not a fit but obtained from the extrapolation scheme discussed above (Eq. 7) using experimental data (Fig. S6). Taken together, the data on DNA-bridging restriction enzymes suggest that we can indeed observe the formation and size of protein-induced topological domains from the reduction of extension fluctuations in the plectonemic regime.

**Table 1.** Two-site restriction enzymes used for DNA bridging measurements.

Enzyme	DNA binding sequence	Loop length Δ*L*
BsaXI	5’-...(N)_9_AC(N)_5_CTCC(N)_10_...-3’	331 bp (113 nm)
NaeI	5’-...GCCGGC...-3’	747 bp (254 nm)
SacII	5’-...CCGCGG...-3’	2683 bp (912 nm)

The enzymes were selected as they possess only two binding sites in the 7.9 kbp sequence used in the MT experiments. The second and third columns give the DNA binding sequence and the predicted loop length calculated from the DNA sequence.

### Distribution and dynamics of topological domains induced by DNA bridging

**Fig. 5.**
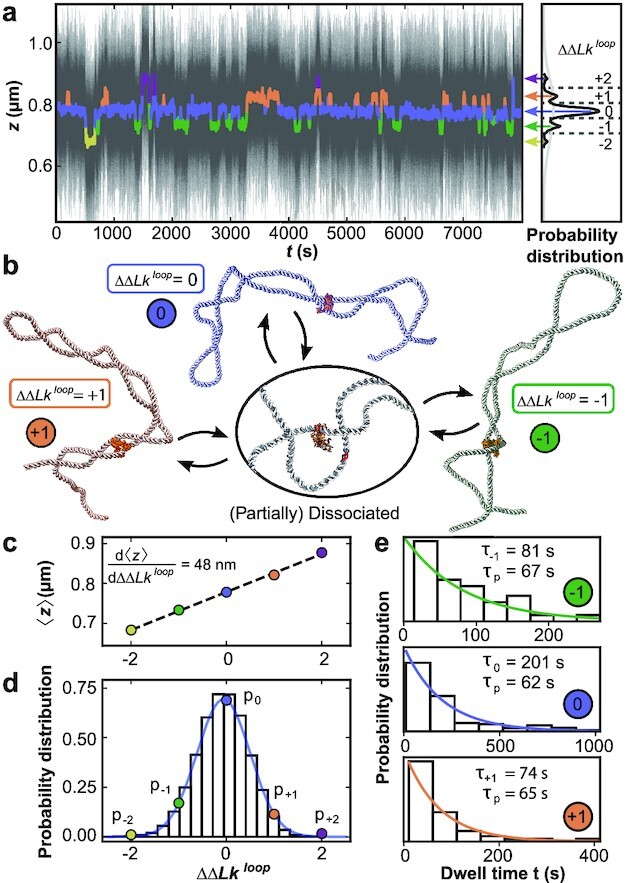
MT measurements reveal the dynamics of topological domains induced by DNA bridging NaeI. (a) Extension time trace in the presence of NaeI in Ca^2 +^ buffer at *f* = 0.5 pN and σ = 0.04. The thin black lines are raw data at 1 kHz. Data filtered by a 10 s sliding average are shown as thick colored lines and exhibit clear transitions between different extension states. Extension states correspond to separate peaks in the extension histogram of the filtered trace on the right and are highlighted using different colors. (b) Illustration of different protein restrained states (crystal structure of NaeI from PDB ID: 1IAW (58)), as identified in panel a: partial protein dissociation and rebinding leads to sampling of states with different ΔΔ*Lk*^loop^ and, consequently, different extensions. (c) Mean extension of the different states assigned in panel a as a function of their relative linking difference ΔΔ*Lk*^loop^. (d) Relative occupancy of the different extension states. The colored dots show the relative occupancy of the different levels observed experimentally and are well described by a Gaussian fit (blue line). The bars show the linking number distribution in plectonemic loops of the same size as the NaeI-induced loop generated with Monte Carlo simulations, with the mean shifted to zero. (e) Dwell time distributions for the three most populated states identified in panel (a). The data are well described by single exponentials (colored lines). The fitted mean dwell times for each state and the implied intrinsic lifetime τ_*p*_ are shown as insets.

Even though the mean DNA extension, 〈*z*〉, for long measurements is essentially unaltered by DNA-bridging restriction enzymes (Fig. 4a and b), closer inspection of the traces reveals transitions between different extension levels, most clearly visible for NaeI. Filtering the raw extension time traces with a 10 s sliding window average highlights transitions between different discrete extension levels (Fig. 5a). The different extension states are separated by 48 ± 2 nm (Fig. 5b and c), which is close to the slope of the extension vs. Δ*Lk* in the plectonemic region for bare DNA under the same condition (53 ± 2 nm). The transitions are thus consistent with integer linking number exchange between the looped and stretched domains that occur during dissociation and rebinding of one or both of the protein binding sites, as illustrated in Fig. 5b. We note that the theory predicts that changes in the extensions should also be accompanied by changes in the variance. However, we estimated these to be too small to be observed in experiments (Supplementary Material Section 2E).

Denoting the extension states by the change in linking number of the looped domain, ΔΔ*Lk*^loop^, relative to the most populated level, we observe changes in linking number of ±2. The relative occupancy of the ΔΔ*Lk*^loop^ states observed in experiments is Gaussian distributed (Fig. 5d, points and blue line) and closely match the linking number distribution obtained from Monte Carlo simulations (Fig. 5d, black bars) generated under the same conditions. The excellent agreement between the two distributions suggests that the relative occupancy of the extension states sampled in the NaeI data are dominated by the supercoiling free energy, while the dissociation and rebinding of NaeI is largely independent of the supercoiling state within the loop. The width of the experimentally determined Gaussian ΔΔ*Lk*^loop^ distribution sampled by NaeI allows us to determine the torsional stiffness of the plectoneme (Supplementary Material Section 2C),

(8) $$\begin{eqnarray*}
P = \frac{\Delta L}{4\pi ^2 \langle \left( \Delta \Delta {Lk}^{\textrm {loop}} \right)^2 \rangle },
\end{eqnarray*}
$$

for which we find *P* = 20 ± 1 nm, where the error was estimated from the covariance matrix of the fit. Our value obtained from the NaeI sampling of linking number states is in good agreement with previously reported estimates of *P* (20,41,59).

In addition to the distribution of ΔΔ*Lk*^loop^ states in the topological domain defined by NaeI binding, the time traces also provide information about the kinetics of the transitions between the states. We use the filtered time traces to obtain dwell time distributions in the different states, and we focus on the three most populated states with ΔΔ*Lk*^loop^ = 0 and ± 1. The dwell time distributions are stochastic and follow single exponential decays (Fig. 5e). The mean lifetimes, τ_*i*_, differ across the different ΔΔ*Lk*^loop^ states (Fig. 5e, insets). A simple stochastic theory (Supplementary Material Section 2D and Fig. S9) suggests that an overall characteristic dissociation time, τ_*p*_, can be obtained as τ_*p*_ = (1 − *p_i_*)τ_*i*_, with *p_i_* the relative occupancy of the states. From the previous relation, we find very similar values for τ_*p*_ for the different ΔΔ*Lk*^loop^ (Fig. 5 e; τ_*p*_ = 65 ± 3 s from the mean and SD of the three most populated states). The timescale τ_*p*_ reflects the dynamics of loop dissociation and reformation. Interestingly, a previous measurement found much longer lifetimes (>1000 s) of loops induced by NaeI in the absence of stretching forces (60), which might suggest that the application of force destabilizes the protein-DNA interfaces.

## Discussion

By combining high-speed MT, Monte Carlo simulations, and analytical theory, we measure and quantitatively describe the extension fluctuations of supercoiled DNA. We use DNA end-to-end fluctuations to monitor topological domain formation by proteins that bridge across two DNA sites. A central result of the paper is Eq. (6), which shows that the variance of the end-to-end distance *z* drops when a topological domain forms by an amount proportional to the domain length, Δ*L*. The proportionality factor can be determined from experimental data by straight-forward extrapolation of the variance of extension fluctuations of bare DNA in the plectonemic regime, Eq. (7) (Fig. S6). Both extensive Monte Carlo simulations and experiments using proteins with unique binding sites closely follow the model prediction. We note that, although other approaches can be used to describe fluctuations in *z* (16,52), the derivation of Eq. (6) relies on the two-phase model description of DNA supercoiling (46).

We anticipate that our methodology to determine the size and dynamics of topological domains from extension fluctuations will provide access to the complex interplay of supercoiled DNA with interacting proteins and co-factors. Conversely, we foresee the opportunity to use sequence-independent bridging proteins to map the size distribution of plectonemes and to identify multiplectoneme phases. Finally, we believe that measurements of end-to-end fluctuations will have an impact beyond supercoiling, in particular in other systems where part of the DNA contour is hidden in a different phase, e.g., in chromatin arrays or protein-induced condensates. The experimental and theoretical framework described in this work is expected to serve as a foundation for a more general adoption of fluctuation analysis in single-molecule force- and torque spectroscopy.

## Methods

DNA construct, experimental procedures, and data analysis. DNA constructs end-labeled with biotin and digoxygenin for MT experiments were prepared as described previously (41). MT measurements were performed on a custom-built instrument (61). Please refer to the Supplementary Materials for details. All experimental results were obtained by video-based tracking at 1 kHz in real-time using a Labview routine (62). Experiments were either performed in phosphate buffered saline (1x PBS buffer; for Fig. 1) or in a buffer comprising 50 mM potassium acetate, 20 mM Tris-acetate, 10 mM calcium acetate, and 100 µg/ml BSA (pH 7.0 at room temperature; for Figs. 4 and 5). Data were evaluated with custom Matlab and Python scripts to deduce force- and linking number-dependencies of extension fluctuations and the effect of protein binding.

Monte Carlo simulations DNA molecules were represented by coarse-grained beads, and conformations of the DNA chain sampled with a Monte Carlo algorithm similar to those used previously (5,63–70). For details, please refer to the Supplementary Materials.

## Supplementary Material

pgac268_Supplemental_File

## Data Availability

The data underlying this article are available freely in the repository YODA and can be accessed at https://doi.org/10.24416/uu01-3jzdap.
